# BUILDING DEMENTIA-FRIENDLY COMMUNITY IN MICHIGAN: THE EXPERIENCES OF BATTLE CREEK

**DOI:** 10.1093/geroni/igz038.1668

**Published:** 2019-11-08

**Authors:** Karla Fales

**Affiliations:** Region 3B Area Agency on Aging, Battle Creek, Michigan, United States

## Abstract

Alzheimer’s disease is now the 6th leading cause of death in Calhoun County – a mostly rural county of 156,000 people in southwest Michigan. In 2017, a cross-sector collaborative launched Dementia Friendly Battle Creek led by CareWell Services Area Agency on Aging. This study focuses on evaluation of the progress and outcomes of this initiative. The goal is to create a community that features high awareness of dementia, coordinated systems of care, and an environment that promotes dignity and engagement of PWD. Using participatory community planning, three Pillars of Impact were developed: Awareness, Coordination, and Environment. More than 500 people have engaged in the effort through trainings, educational programs, expanded supportive services, and the creation of integrated pathways of care for PWD. Additionally, more than $100,000 has been leveraged to support the effort. Challenges included sustaining momentum & gaining buy-in from local municipalities and health care providers.

